# Satralizumab Ameliorates Refractory Central Neuropathic Pain and Painful Tonic Spasms in Neuromyelitis Optica Spectrum Disorder: A Case Report

**DOI:** 10.7759/cureus.86335

**Published:** 2025-06-19

**Authors:** Junichi Matsuo, Keiichi Nakahara, Yasuyuki Hara, Mitsuharu Ueda

**Affiliations:** 1 Neurology, Kumamoto Rosai Hospital, Yatsushiro, JPN; 2 Neurology, Kumamoto University, Kumamoto, JPN

**Keywords:** central neuropathic pain, interleukin-6, neuromyelitis optica spectrum disorder, painful tonic spasm, satralizumab

## Abstract

Neuromyelitis optica spectrum disorder (NMOSD) is frequently accompanied by severe central neuropathic pain and painful tonic spasms for which therapeutic options are limited. Satralizumab, a humanized monoclonal antibody targeting the interleukin-6 receptor, has reduced relapse rates among patients with anti-aquaporin-4 antibody-positive NMOSD. Although reports have suggested that satralizumab may reduce NMOSD-associated central neuropathic pain and painful tonic spasms, conclusive evidence does not yet exist. Herein, we describe a patient with NMOSD whose refractory central neuropathic pain and painful tonic spasms did not adequately respond to conventional treatments but markedly improved following the initiation of satralizumab. This case suggests that satralizumab may be effective for central neuropathic pain and painful tonic spasms associated with NMOSD and could be an alternative therapy when other treatments are ineffective.

## Introduction

Neuromyelitis optica spectrum disorder (NMOSD) is a severe autoimmune astrocytopathy characterized by recurrent inflammation that predominantly affects the optic nerves, brain, and spinal cord [1]. Interleukin-6 (IL-6) is a pleiotropic cytokine with multiple functions, with roles in acute and chronic inflammation, host defense mechanisms, hematopoiesis, and synthesis of acute-phase proteins. Dysregulated and persistent production of IL-6 has been implicated in the pathogenesis of several autoimmune diseases [2]. In NMOSD, damage to the central nervous system occurs primarily through the binding of anti-aquaporin 4 (AQP4) antibodies, which activate the complement pathway, leading to neuronal cell death. IL-6 promotes the production of anti-AQP4 antibodies and increases the permeability of the blood-brain barrier, making it a crucial target for NMOSD treatment [3]. Clinical trials have demonstrated that satralizumab, an IL-6 inhibitor, has excellent efficacy in preventing NMOSD relapse; however, its role in alleviating NMOSD-associated central neuropathic pain and painful tonic spasms has not been established [4,5].

Herein, we report the case of a Japanese patient with refractory central neuropathic pain and painful tonic spasms secondary to AQP4-positive NMOSD, whose symptoms were relieved following the initiation of satralizumab after the failure of conventional immunotherapies.

## Case presentation

A 69-year-old Japanese man was admitted to our hospital with the chief complaint of abnormal limb sensation in his hands and feet for a month, which had worsened 2 days before.

On admission, the patient was alert and oriented. His vital signs were as follows: body temperature, 36.7 °C; blood pressure, 127/83 mmHg; and pulse rate, 83 beats per minute, with a regular rhythm. Neurological examination revealed no abnormalities in the cranial nerves but muscle weakness in the left upper limb, bilateral (more pronounced on the left) decreased superficial and proprioceptive sensations in the limbs and trunk, hyperreflexia in the limbs, and urinary retention. Pain from the back to the lower limbs was rated 10/10 on a numerical rating scale (NRS). Laboratory tests showed that the serum anti-AQP4 antibody was positive by enzyme-linked immunosorbent assay, while other autoantibodies were negative.

**Figure 1:**
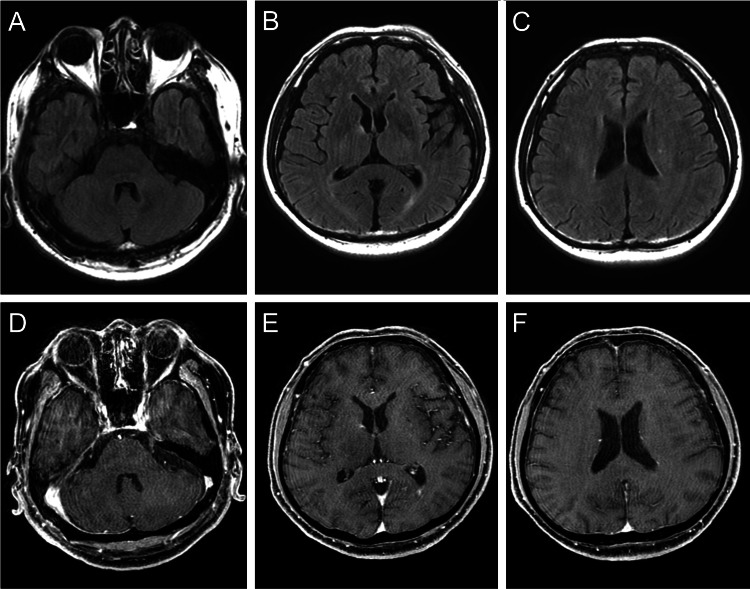
Brain MRI on admission No apparent abnormalities are observed on FLAIR imaging (A-C) or Gadolinium-enhanced T1-weighted imaging (D-F). MRI, magnetic resonance imaging; FLAIR, fluid-attenuated inversion recovery

Analysis of the cerebrospinal fluid revealed pleocytosis (mononuclear cells: 146/μL, polymorphonuclear cells: 32/μL) and an elevated total protein level of 218 mg/dL. The immunoglobulin G (IgG) index was 0.62, and intrathecal oligoclonal bands were negative. Contrast-enhanced brain magnetic resonance imaging (MRI) findings were unremarkable (Figure 1).

**Figure 2:**
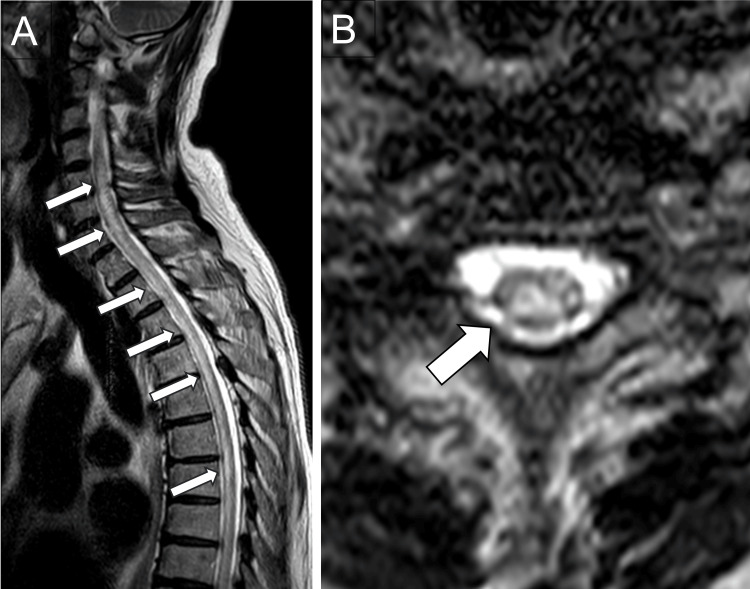
Spinal MRI on admission Sagittal (A) and axial (B) T2-weighted images show an extensive longitudinal transverse myelitis lesion (arrows) in the spinal column from the upper cervical to the thoracic region. MRI, magnetic resonance imaging

Whole-spine contrast-enhanced MRI revealed high signal areas on T2-weighted images extending from C3 to Th10 without contrast-enhanced lesions (Figure 2).

Given the positivity of serum anti-AQP4 antibodies and acute myelitis and the exclusion of other diseases, the patient was diagnosed with anti-AQP4 antibody-positive NMOSD.

**Figure 3:**
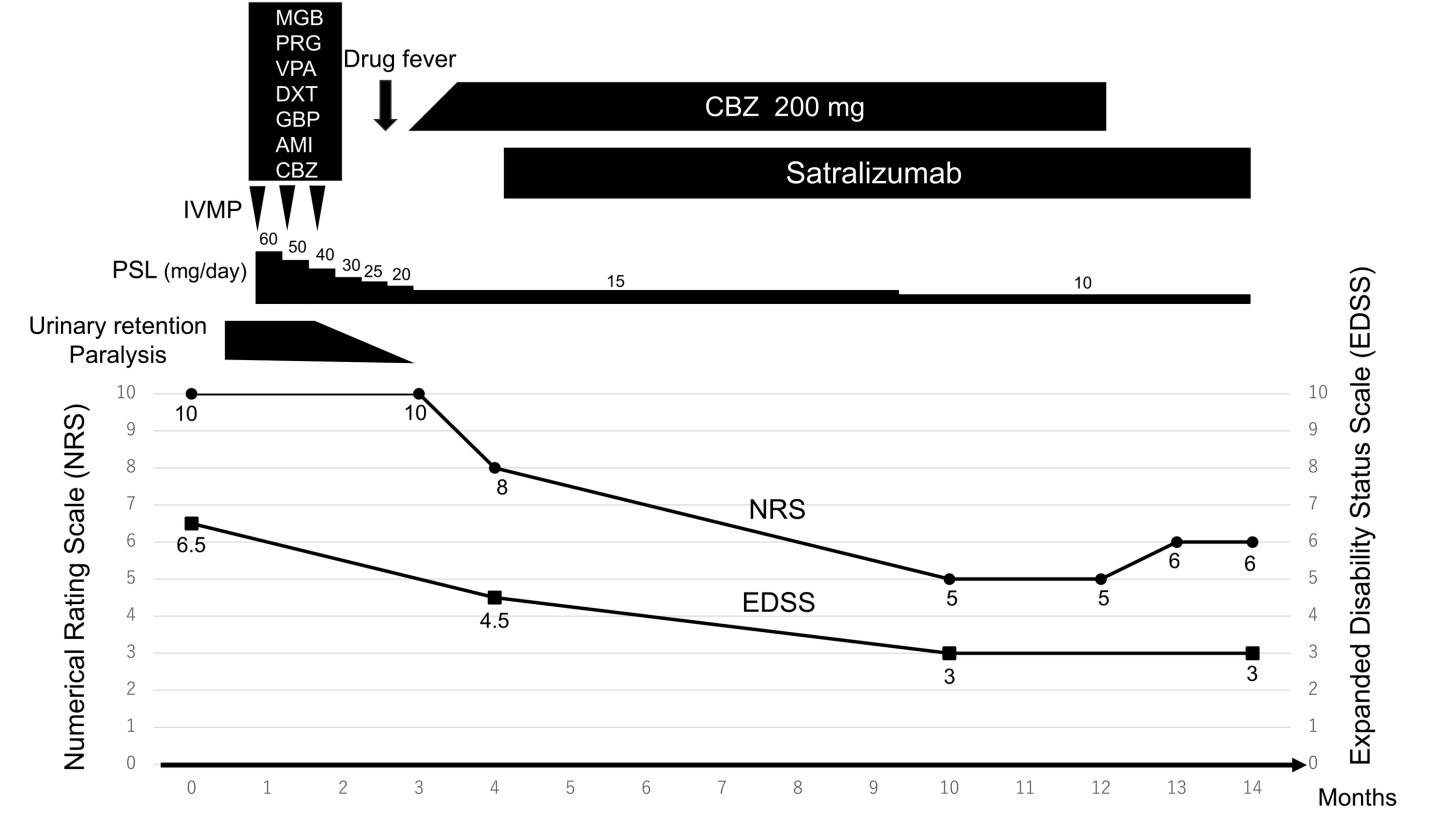
Clinical course of the present case Central neuropathic pain, which was unresponsive to treatment with multiple analgesics, IVMP, or oral PSL, gradually improved after the initiation of combination therapy with CBZ and satralizumab. During hospitalization, the patient received various analgesics with the following administration periods: MGB was administered from hospital days 31 to 84, PGB from 85 to 92, VPA from 70 to 81, DXT from 74 to 81, GBP from 81 to 91, and AMI from 81 to 91. After one year of treatment, CBZ was successfully discontinued, with only mild residual pain that did not affect the patient's daily life. IVMP, intravenous methylprednisolone; PSL, prednisolone; MGB, mirogabalin; PGB, pregabalin; VPA, valproate; DXT, duloxetine; GBP, gabapentin; AMI, amitriptyline; CBZ, carbamazepine; NRS, numerical rating scale; EDSS, expanded disability status scale

The clinical course of the patient is summarized in Figure 3.

Following admission, he was administered intravenous methylprednisolone (1,000 mg/day for 5 days) followed by oral prednisolone (60 mg/day). Prednisolone was tapered gradually on a weekly basis. Subsequently, two additional intravenous methylprednisolone courses (1,000 mg/day for 3 days) were administered. Overall, the symptoms improved, but painful abnormal sensations from the back to the lower limbs (NRS score: 10/10) and painful tonic spasms persisted. Painful tonic spasms were evaluated based on the clinical presentation of radiating pain that occurs paroxysmally in both lower limbs and lasts for several seconds to several minutes. Various medications, including mirogabalin, valproate, duloxetine, gabapentin, pregabalin, and amitriptyline, were initiated for central neuropathic pain and painful tonic spasms; however, none were effective because there was no subjective improvement in the severity of pain or painful tonic spasms. Carbamazepine (CBZ) was the only effective medication for painful tonic spasms but was temporarily discontinued due to drug-induced fever. As the painful tonic spasms worsened, CBZ was reintroduced at a low dose and gradually increased, with no recurrence of the drug-induced fever. Three months after the disease onset, the patient was stable on CBZ at a dose of 200 mg/day. Although the painful tonic spasms eased, the central neuropathic pain persisted (NRS: 8/10). Four months after the disease onset, satralizumab was introduced to prevent relapse. Following the initiation of satralizumab, the central neuropathic pain from the back to the lower limbs gradually improved, showing substantial improvement 10 months after the onset (NRS: 5/10). One year after the disease onset, CBZ was discontinued because of an improvement in pain and concerns about potential side effects. After discontinuation, the central neuropathic pain worsened slightly (NRS: 6/10) but did not fully return to its previous severity and did not affect activities of daily life.

## Discussion

Central neuropathic pain and nociceptive pain are prominent and debilitating symptoms of NMOSD, affecting approximately three-quarters of the patients. With standard therapies, more than 60% of the patients continue to experience moderate-to-severe pain [6]. The underlying mechanisms include spinal microglial activation and dysfunction of descending inhibitory pathways, which are exacerbated by mood disturbances [6,7]. These pain symptoms greatly impair the quality of life, and conventional neuropathic pain agents, such as gabapentinoids, tricyclic antidepressants, and serotonin-norepinephrine reuptake inhibitors (SNRIs), frequently provide only partial relief [8]. Since there are no randomized controlled trials targeting pain in NMOSD, this case was treated in accordance with approaches used for neuropathic pain and pain caused by multiple sclerosis [9,10].

IL-6 is central to both NMOSD pathogenesis and the development of central neuropathic pain [11]. It drives the differentiation of naïve T cells into Th17 effectors, promotes the maturation of B cells into plasmablasts (thereby amplifying anti-AQP4 antibody production), and disrupts the integrity of the blood-brain barrier, facilitating the infiltration of immune cells into the central nervous system [3,12-14]. Preclinical studies in mice with experimental autoimmune encephalomyelitis have shown that blocking the IL-6 receptor suppresses microglial activation, reduces pro-inflammatory mediators in the spinal cord, and restores descending pain inhibition, resulting in sustained analgesia [15,16].

Observational studies and case series have supported the analgesic potential of IL-6 receptor antibodies. Tocilizumab, a chimeric anti-IL-6 receptor antibody, reduced central neuropathic pain and fatigue in a small group of patients with refractory NMOSD [17]. Satralizumab, a humanized anti-IL-6 receptor monoclonal antibody approved for the prevention of NMOSD relapses, has demonstrated robust relapse reduction in Phase III trials. However, it did not show a statistically significant effect on pain or fatigue as secondary endpoints, possibly because of limited statistical power and prioritization of secondary endpoints [4,5]. However, individual case reports have documented striking analgesic effects. For example, Uzawa et al. described the complete resolution of painful tonic spasms after six months of satralizumab treatment in a patient with anti-AQP4 antibody-positive NMOSD [18]. Recently, a patient with refractory NMOSD who had previously been treated with inebilizumab experienced substantial relief from central neuropathic pain following the initiation of satralizumab treatment [19]. These reports demonstrate pain improvement occurring several months following the initiation of satralizumab, which closely resembles the clinical course observed in our case. Furthermore, IL-6 inhibition may be effective for lower back pain caused by intervertebral disc degeneration and pain associated with CASPR2 antibody-related autoimmune encephalitis [20,21].

Emerging case reports, including ours, suggest that an IL-6 blockade may provide additional benefits beyond relapse prevention in patients with NMOSD. This is likely due to the attenuation of microglia-mediated neuroinflammation and normalization of abnormal neuronal excitability. Our patient experienced a gradual yet sustained reduction in central neuropathic pain (NRS score falling from 8/10 to 5/10 over 6 months) and an end to painful tonic spasms despite the cessation of additional anticonvulsants. Together with prior anecdotal evidence, this report underscores the need for a systematic investigation of the impact of satralizumab on pain outcomes in NMOSD. Furthermore, it has been reported that the presence of upper thoracic spinal cord lesions and the frequency of recurrence of myelitis are associated with refractory pain in NMOSD. Patients with these characteristics may potentially benefit from the analgesic effects of an IL-6 blockage [6]. However, the limitations include the single-patient design, lack of objective pain biomarkers, the natural course of the disease, and potential confounding factors of concomitant medications. Although CBZ may have played a partial role in pain improvement, the observation that pain levels did not deteriorate to the pre-satralizumab baseline following the discontinuation of CBZ further supports the involvement of satralizumab in alleviating pain. Prospective studies involving detailed pain phenotyping and objective biomarkers (e.g., painDETECT scores, quantitative sensory testing, functional MRI) are required to determine the role of IL-6 inhibition in NMOSD-associated central neuropathic pain and painful tonic spasms [22,23].

## Conclusions

This case adds to the growing body of evidence suggesting that satralizumab alleviates refractory central neuropathic pain and painful tonic spasms in patients with NMOSD. These findings highlight the potential of an IL-6 blockade as a promising treatment for these debilitating symptoms. Further controlled trials are essential to validate these preliminary observations and to optimize pain management in patients with NMOSD.
